# Axonemal Growth and Alignment During Paraspermatogenesis in the Marine Gastropod *Strombus luhuanus*

**DOI:** 10.3389/fcell.2022.905748

**Published:** 2022-06-27

**Authors:** Daisuke Shibata, Masaya Morita, Yu Sato, Kogiku Shiba, Seiya Kitanobo, Ryo Yokoya, Kazuo Inaba

**Affiliations:** ^1^ Shimoda Marine Research Center, University of Tsukuba, Shizuoka, Japan; ^2^ Sesoko Station, Tropical Biosphere Research Center, University of the Ryukyus, Okinawa, Japan

**Keywords:** parasperm, spermatogenesis, cilia, axoneme, dynein, fertilization, gastropod

## Abstract

Parasperm are non-fertilizing sperm that are produced simultaneously with fertile eusperm. They occur in several animal species and show considerable morphological diversity. We investigated the dynamics of axonemes during paraspermatogenesis in the marine snail *S. luhuanus*. Mature parasperm were characterized by two lateral undulating membranes for motility and many globular vesicles. Axonemes were first observed as brush-like structures that extruded from the anterior region. Multiple axonemes longer than the brush then started to extend inside the cytoplasm towards the posterior region. The mass of the axonemes separated into two lateral rows and formed an undulating membrane that drives bidirectional swimming in the mature parasperm. The central pair of axonemes was aligned in the undulating membrane, resulting in cooperative bend propagation. During paraspermatogenesis, centrioles were largely diminished and localized to the anterior region. CEP290, a major component of the transition zone, showed a broad distribution in the anterior area. Axonemes in the posterior region showed a 9 + 0 structure with both outer and inner arm dyneins. These observations provide a structural basis for understanding the physiological functions of parasperm in marine reproductive strategies.

## Introduction

Sperm heteromorphism occurs in most animal phyla. Parasperm are formed in the testis, but in contrast to eusperm, do not participate in fertilization. Parasperm that lack some or all chromosomal DNA are referred to as oligopyrene or apyrene sperm, respectively (Buckland-Nicks, 1998; Hayakawa, 2007). The simultaneous formation of parasperm in the limited testicular space is thought to reflect their essential roles in male reproductive strategies, such as transport of eusperm, protection of eusperm from toxic substances in the female tract, sperm competition, and supplying of nutrients to the eusperm. For example, parasperm protect eusperm from spermicidal activity in the female reproductive tract of the fruit fly *Drosophila pseudoobscura* (Holman and Snook, 2006). In the marine sculpin, *Hemilepidotus gilbert*, parasperm form a lump structure to prevent fertilization by rival eusperm (Hayakawa et al., 2002). A comprehensive understanding of the structure and function of parasperm could help elucidate the reproductive strategies that animals have acquired during evolution in response to complex environmental strains.

Prosobranchia, a large group of marine, land, and freshwater snails, show a variety of parasperm morphologies (Reinke, 1912; Fretter, 1953; Ishizaki and Kato, 1958; Tanaka, 1958; Yasuzumi and Tanaka, 1958; Yasuzumi et al., 1960; Gall, 1961; Nishiwaki, 1964; Koike and Nishiwaki, 1980; Melone et al., 1980; Jamieson, 1987; Koike and Shinohara, 1987; Healy and Jamieson, 1993; Hodgson, 1997; Buckland-Nicks, 1998; Buckland-Nicks et al., 2000; Hayakawa, 2007; Higginson and Pitnick, 2011). Because of this diversity, parasperm have been extensively used in the taxonomic description of this group. Among the several types of parasperm in this subclass, those in the superfamily Stromboidea show remarkable morphological characteristics, including large granular structures and two peripheral undulating membranes with numerous axonemes. Light and transmission electron microscopy have shown changes in the cytoplasmic structure during paraspermatogenesis, including degeneration of the nucleus and formation of granular structures and undulating membranes (Brock, 1887; Reinke, 1912; Koike and Nishiwaki, 1980; Buckland-Nicks, 1998; Buckland-Nicks et al., 2000). However, little is known about the mechanism of formation and movement in the undulating membranes and how the axonemes are arrayed within.

In this study, we first investigated the changes in axonemal localization during parasperm formation in the marine snail *S. luhuanus*. Second, we examined the wave propagation of undulating membranes and its associations with the orientation of axonemal growth and alignment. We evaluated the anterior/posterior position of the mature parasperm and its relationship to motility. We also described a unique change in the distribution of basal bodies and the transition zone during spermatogenesis.

## Materials and Methods

### Experimental Materials

*S. luhuanus* were collected near Shimoda Marine Research Center (Shimoda, Shizuoka) from May to September in 2009–2020, and near Sesoko Station of the University of the Ryukyus (Motobu, Okinawa) from February to March in 2014 and 2022. Mature sperm or spermatogenic cells were obtained from the male sperm duct or testis, respectively, and suspended in artificial seawater (ASW) consisting of 460.3 mM NaCl, 10.11 mM KCl, 9.18 mM CaCl_2_, 35.91 mM MgCl_2_, 17.49 mM MgSO_4_, 0.1 mM EDTA, and 10 mM Hepes-NaOH (pH 8.2).

### Observations of Morphology by Light Microscopy

Sperm were observed under a differential interference contrast (DIC) microscope (BX51, Olympus, Tokyo, Japan). For the testes, a section was fixed in Bouin’s solution at room temperature overnight. The samples were dehydrated using a graded ethanol series and embedded in paraffin wax. The paraffin blocks were trimmed and cut into 8-μm-thick sections. The sections were then deparaffinized, rehydrated, and stained with hematoxylin and eosin (H&E).

### Scanning Electron Microscopy

Suspensions of testicular cells or mature sperm from the sperm duct were immobilized on a poly L-lysine-coated coverslip. Samples were fixed in 2.5% glutaraldehyde in 0.45 M sucrose and 0.1 M sodium cacodylate (pH 7.4) at 4°C for 1 h. Fixed samples were washed three times with 0.1 M sodium cacodylate (pH 7.4), dehydrated using a graded ethanol series, substituted with t-butyl alcohol and freeze-dried (JFD-320, JEOL, Tokyo, Japan), coated with Au using an ion sputter gun, and observed under a scanning electron microscope (NeoScope JCM-5000, JEOL).

### Transmission Electron Microscopy

Testes and sperm duct sections were observed by transmission electron microscopy (TEM), according to a previously described protocol (Konno et al., 2010). Briefly, trimmed pieces of testis were fixed with 2.5% glutaraldehyde in 0.45 M sucrose, 0.1 M sodium cacodylate (pH 7.4), and washed with 0.1 M sodium cacodylate (pH 7.4). The fixed samples were post-fixed with 1% OsO_4_ at 4°C for 1 h. After dehydration in a graded ethanol series, samples were embedded in Quetol 812 (Nisshin EM, Tokyo, Japan) and solidified at 60°C for 48 h. Sections were made using an ultramicrotome at an average thickness of 70 nm. The sections were stained with uranyl acetate and lead citrate and observed under a transmission electron microscope (JEM 1200EX, JEOL).

### Antibodies

Mouse polyclonal antibody against CEP290 was prepared as previously described (Padma et al., 2003). The primers used to amplify *S. luhuanus* CEP290 cDNA were 5′-GCGCGGATCC ATG GAA CTT CGT TTT GAG-3′ (forward) and 5′-GCGCGAATTC CTA TAT ACC GGG TAC ACC-3′ (reverse). An antibody against PF16 (known as SPAG6, a mammalian ortholog) from the ascidian *Ciona intestinalis* was prepared according to the method of Satouh and Inaba (2009). Other antibodies used in this study included anti-γ-tubulin (ab11316, Abcam, Tokyo, Japan; T6557, Sigma-Aldrich, St. Louis, MO, United States), anti-acetylated α-tubulin (mouse IgG, 05-829, Sigma-Aldrich, St. Louis, MO, United States; rabbit, 5335, Cell Signaling Technology, Danvers, MA, United States), anti-β-actin (sc-47778, Santa Cruz Biotechnologies, Santa Cruz, CA, United States), anti-mouse HRP-conjugated secondary (62-6520, Thermo Fisher Scientific, Waltham, MA, United States); anti-rabbit HRP-conjugated secondary (65-6120, Thermo Fisher Scientific, Waltham, MA, United States) anti-mouse secondary (Alexa Fluor 488-conjugated, 11001, Thermo Fisher Scientific; Alexa Fluor 546-conjugated, 11003, Thermo Fisher Scientific); and anti-rabbit secondary antibody (Alexa Fluor 488-conjugated, 11008, Thermo Fisher Scientific). For western blotting, trimmed pieces of testes or mature sperm from the sperm duct were suspended in ASW, and a 5× sample buffer for SDS-PAGE was added to the suspension and boiled for 2 min. Proteins were separated by SDS-PAGE and transferred to polyvinylidene difluoride membranes. The membranes were treated with a blocking buffer containing 7.5% skim milk in PBS containing 0.05% Tween 20 (PBST) for 1 h and then incubated with primary antibodies at a 1:2,000–10,000 dilution for 1 h at room temperature. After washing with PBST, the blots were incubated with the anti-mouse HRP-conjugated secondary antibody at a 1:10,000 dilution for 30 min at room temperature. After washing with PBST three times, the blots were developed using an enhanced chemiluminescence kit (ECL Prime, GE Healthcare, IL, United States).

### Immunofluorescence Microscopy

Immunofluorescence microscopy was performed as previously reported, with some modification (Padma et al., 2003). A suspension of spermatogenic cells or mature sperm was immobilized onto a coverslip coated with poly-L-lysine and fixed with cold methanol at −30°C for 30 min or with 4% PFA in 0.1 M HEPES-NaOH (pH 7.4) and 0.4 M sucrose at room temperature for 1 h. The samples were washed with PBST and incubated in blocking solution (10% goat serum and 1% BSA in PBS) for 1 h at room temperature, followed by incubation with anti-acetylated-α-tubulin (T6793, Sigma-Aldrich, MD, United States), anti-γ-tubulin, anti-CEP290, and anti-β-actin antibody at 1:500 to 1:1,000 dilutions in the blocking solution for 2 h at 4°C. After washing with PBST at room temperature, the samples were incubated with Alexa 488 goat anti-mouse IgG and Alexa 546 goat anti-rabbit IgG or Alexa 488 goat anti-rabbit IgG and Alexa 546 goat anti-mouse IgG at a 1:1,000 dilution in blocking solution for 1 h at room temperature. The samples were washed with PBST at room temperature and incubated with 10 μM 4′,6-diamidino-2-phenylindole (DAPI) in PBS. Images were obtained using an Fv10 confocal microscope (Olympus).

### Analysis of Sperm Motility

Mature sperm from the sperm duct or the suspension of testicular cells were diluted 100–2,000-fold with ASW, placed on a glass slide coated with 1% BSA to avoid adhesion of sperm, and observed under a DIC microscope (BX51or 53, Olympus) with a 10× or 20× objective (UPlanSApo, Olympus). Photographs were obtained using a digital camera (Olympus DP70 or DP74). For video recording at 200 fps, the microscope was connected to a high-speed CCD camera (HAS220 or HASU-2, Ditect, Tokyo, Japan). Data are presented as mean ± SD, unless otherwise stated.

## Results

### Testicular Structure in *S. luhuanus*

**FIGURE 1:**
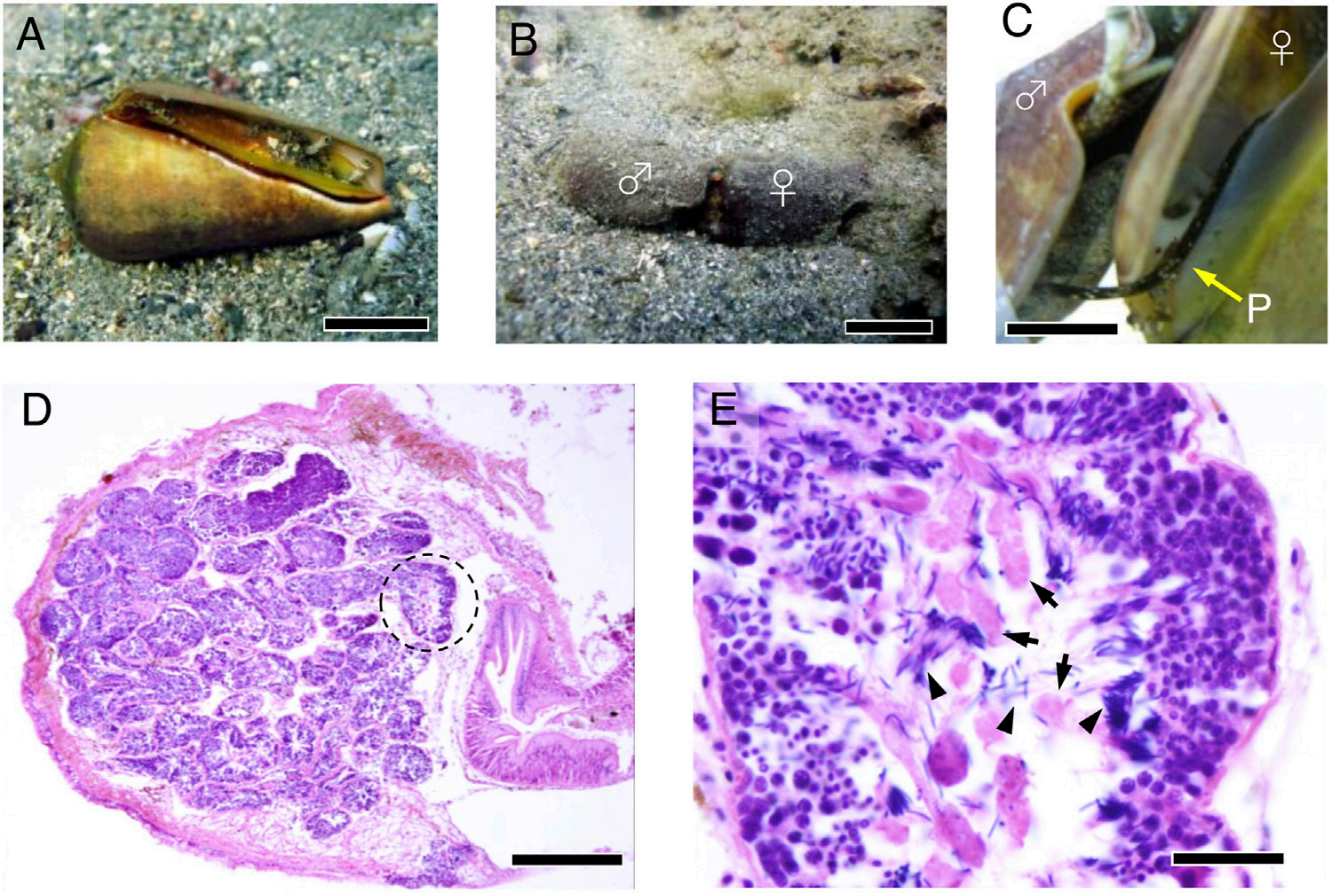
Male reproductive system in *S. luhuanus*. **(A)** Adult *S. luhuanus.* Scale bars, 2 cm. **(B)** A copulating male and a female. Scale bars, 2 cm. **(C)** Insertion of the penis into the female tract. Scale bars, 1 cm. **(D)** H&E staining of *S. luhuanus* testis, which is clearly composed of many lobules (dashed line). Scale bars, 500 μm. **(E)** Both eusperm and parasperm are generated in a lobule. Parasperm and paraspermatogenic cells (arrows) are recognized as larger cells with less intense hematoxylin staining. Spermatids and mature sperm are recognized by packed nuclei intensely stained with hematoxylin (arrowheads). Scale bars, 100 μm.

**FIGURE 2:**
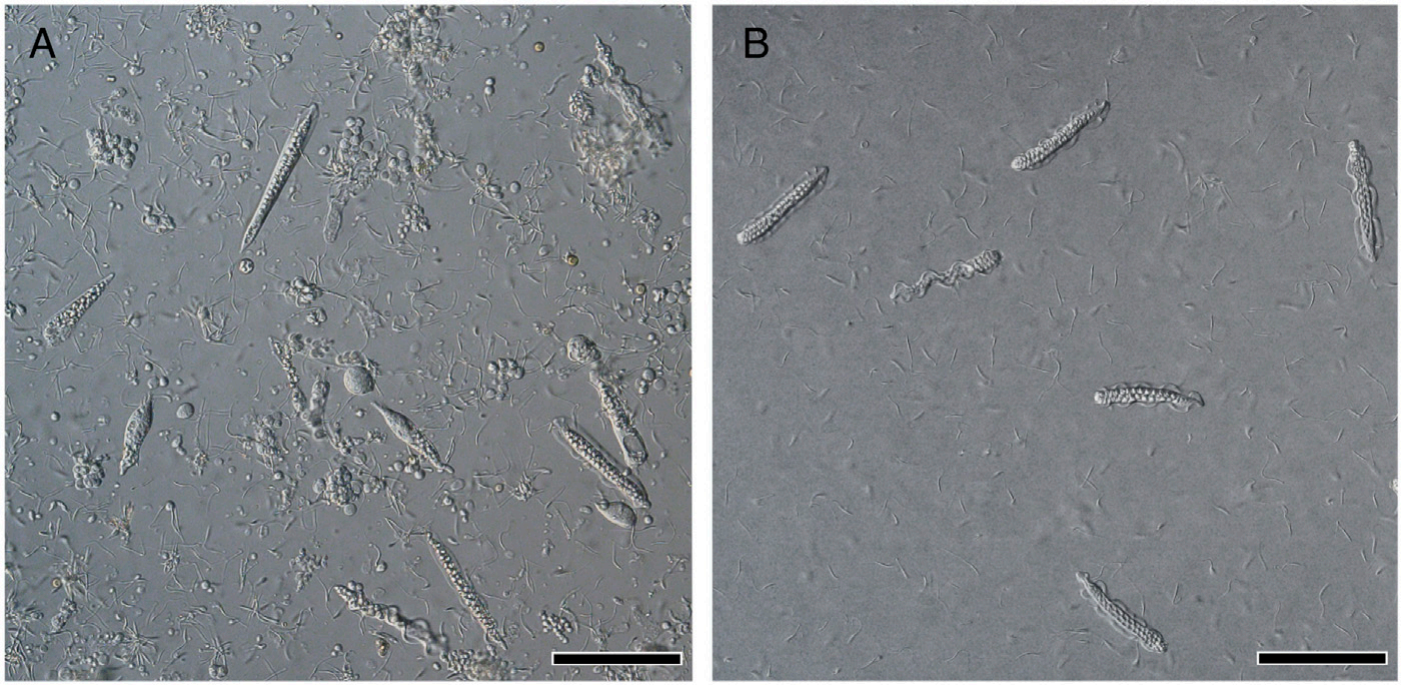
Differential interference contrast (DIC) images of *S. luhuanus* sperm. **(A)** Spermatogenic cells obtained from testis. Both immature eusperm and parasperm can be observed. Scale bar, 100 μm. **(B)** Mature sperm obtained from sperm duct. Six mature parasperm as well as many mature eusperm are shown. Scale bar, 100 μm.

*S. luhuanus* occurs in the sandy substrates near rocky marine areas (Figure 1A). They are gonochoristic, undergo internal fertilization, and reproduce during summer at Shimoda, Shizuoka (temperate zone) and winter at Sesoko, Okinawa (subtropical zone). Males copulate with females and release sperm by intromitting the penis to the female genital tract (Figures 1B,C). Prior to reproductive season, the development of eusperm and parasperm is initiated in the testes. Mature sperm are spermiated into the sperm duct (spermiduct or vas deferens), while spermatogenesis continues in the testis. Eusperm and parasperm were found in separate areas along with spermatogenic cells in testicular lobes without a discrete boundary (Figures 1D,E). In this study, we collected mature sperm and spermatogenic cells from the sperm ducts and testes, respectively, during the reproductive season. Sperm ducts predominantly contained mature eusperm and parasperm in the mid-reproductive season, whereas several spermatogenic cell stages were still observed in the testis (Figure 2).

### Morphology and Motility of Parasperm

**FIGURE 3:**
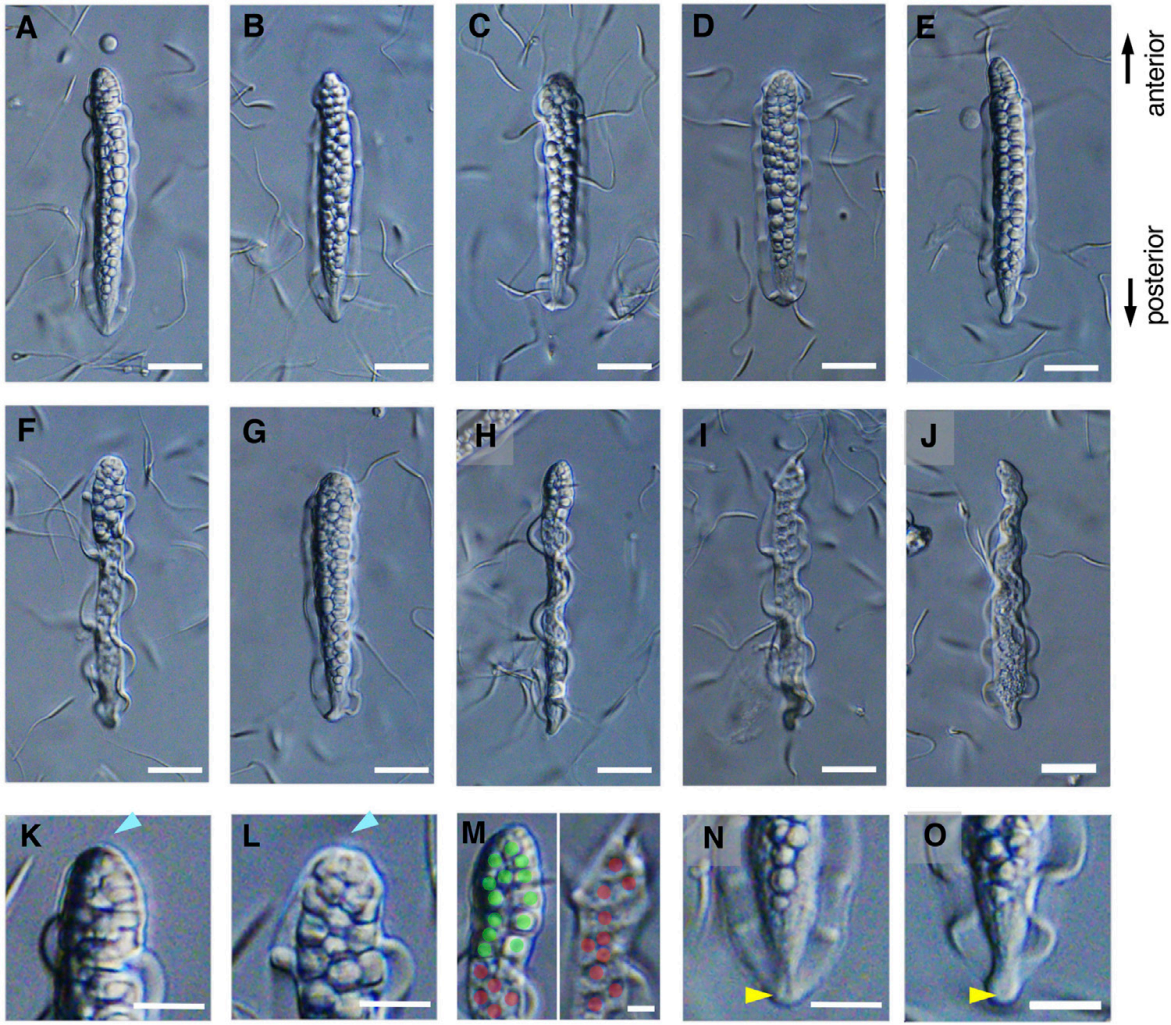
**(A–G)** The mature parasperm, including **(H)** a lateral view. Scale bar, 20 μm. **(I,J)** Some parasperm lacked globular bodies in the sharpened anterior end. Scale bar, 20 μm. **(K,L)** Typical parasperm have a round anterior edge with globular bodies. **(M)** Magnified anterior regions of **(H)** and **(I)** showing partial or complete loss of globular bodies. Green or red represents occupied or empty area for the globular bodies, respectively. Scale bar, 10 μm. **(N,O)** A posterior edge with a flattened end and streak structure. Scale bar, 20 μm.

**FIGURE 4:**
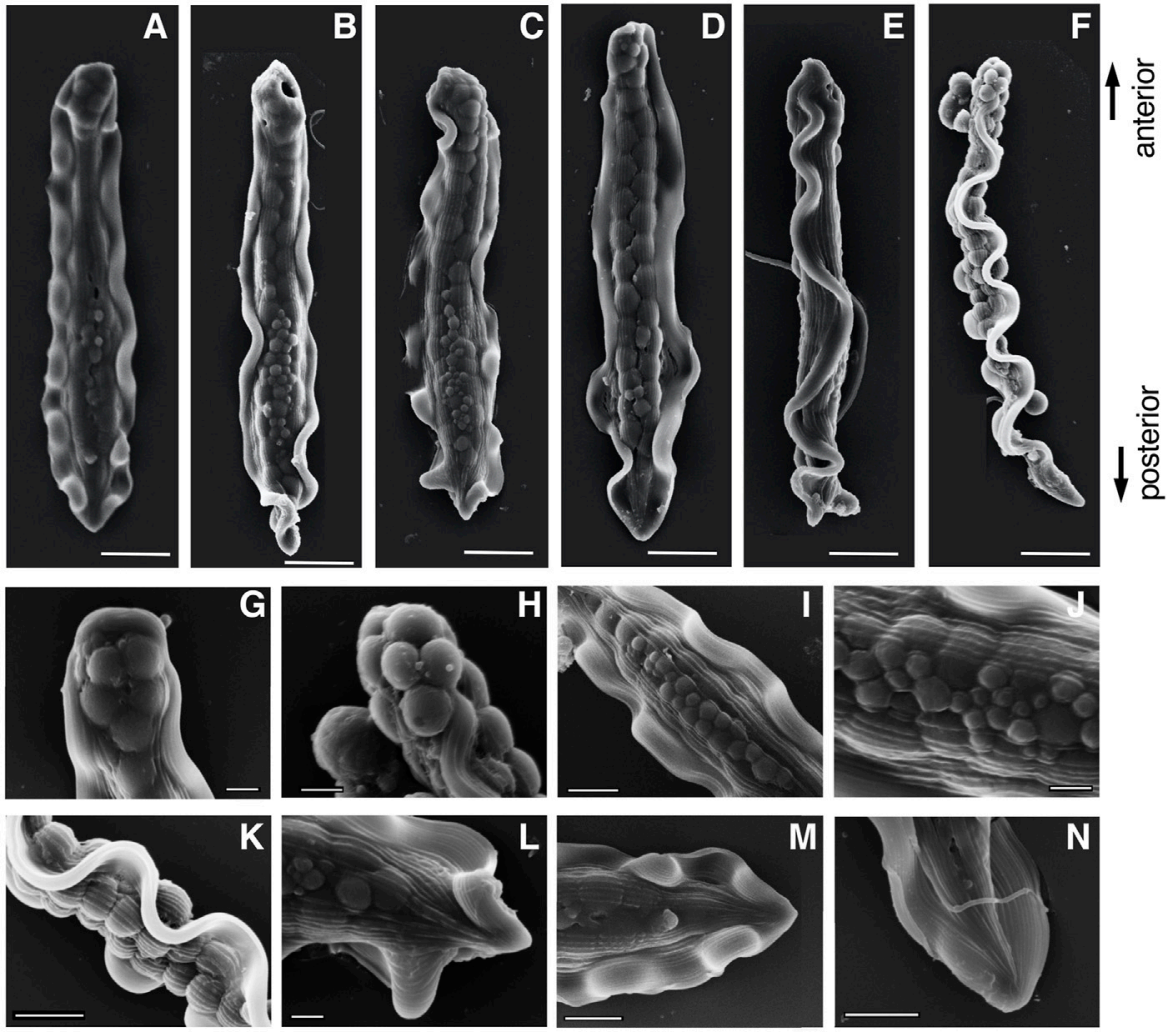
Scanning electron microscope (SEM) images of **(A–F)** entire mature parasperm and **(G–N)** magnified images of distinct morphological regions. **(F)** Lateral view, **(G,H)** anterior round edge, **(I–K)** central areas with globular bodies, and **(L–M)** posterior streaked end. Scale bar in **(A–F)**, 10 μm; **(G–H,J,L)**, 2 μm; and **(I,K,M,N)**, 5 μm.

The mature parasperm of *S. luhuanus* has a unique morphology, with large globular bodies and two lateral undulating membranes (Figures 3A–J, 4A–F; Reinke, 1912; Koike and Nishiwaki, 1980; Casse et al., 1994). Parasperm collected from the sperm duct had globular bodies distributed throughout the cytoplasm. The tip of the cell showed a round edge with tightly packed globular bodies (Figures 3K,L, 4G,H), though a few parasperm appeared to have lost the globular bodies and their tips appeared slender or sharp (Figures 3I,J). The losses of globular bodies were easily recognized by empty spherical regions where globular bodies would have been present (Figure 3M). Two undulating membranes, which are made of a hundred axonemes representing a plate-shaped motile apparatus (Koike and Nishiwaki, 1980; Casse et al., 1994), are laterally located. Several arrays of single axonemes, distinct from the axonemes in the undulating membranes, were observed covering the area of the globular bodies (Figures 4H–K). At the opposite tip, the undulating membranes were joined to form a flattened end with a central “streak” along the longer axis of the parasperm (Figures 3N,O, 4L–N). Following the description in previous reports (Nishiwaki, 1964; Koike and Shinohara, 1987; Buckland-Nicks, 1998), we defined the anterior and posterior ends of the *Strombus* parasperm as those with a round end and streak, respectively (Figures 3, 4, indicated at the right).

**FIGURE 5:**
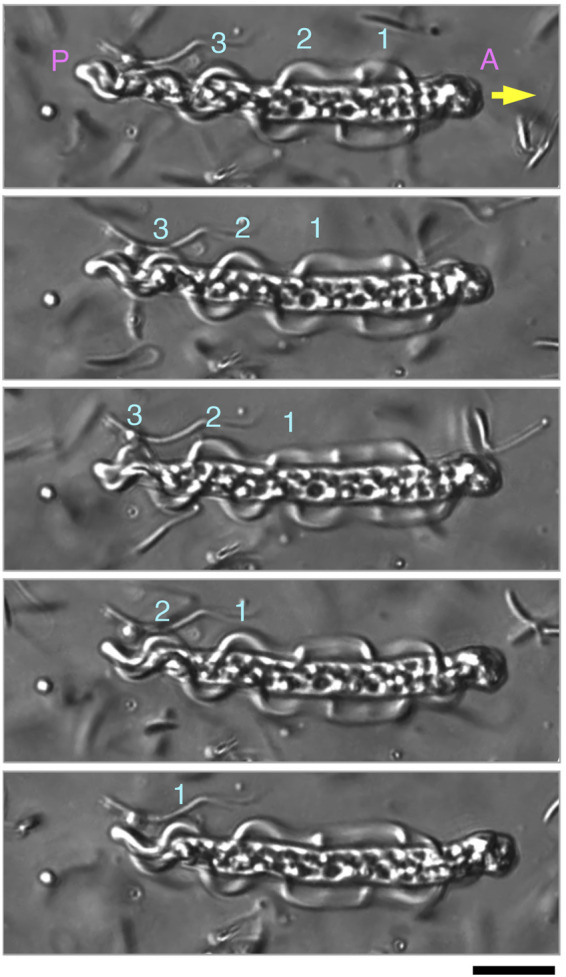
Sequential images of parasperm (top to bottom) recorded at 200 fps in a forward swimming motion (yellow arrow). Images at 300 ms intervals are shown. Three waves (numbers above parasperm) propagated in the undulating membrane from the anterior to posterior end. (A) Anterior end and (P) posterior end. See Supplementary Video S1. Scale bar, 20 μm.

**FIGURE 6:**
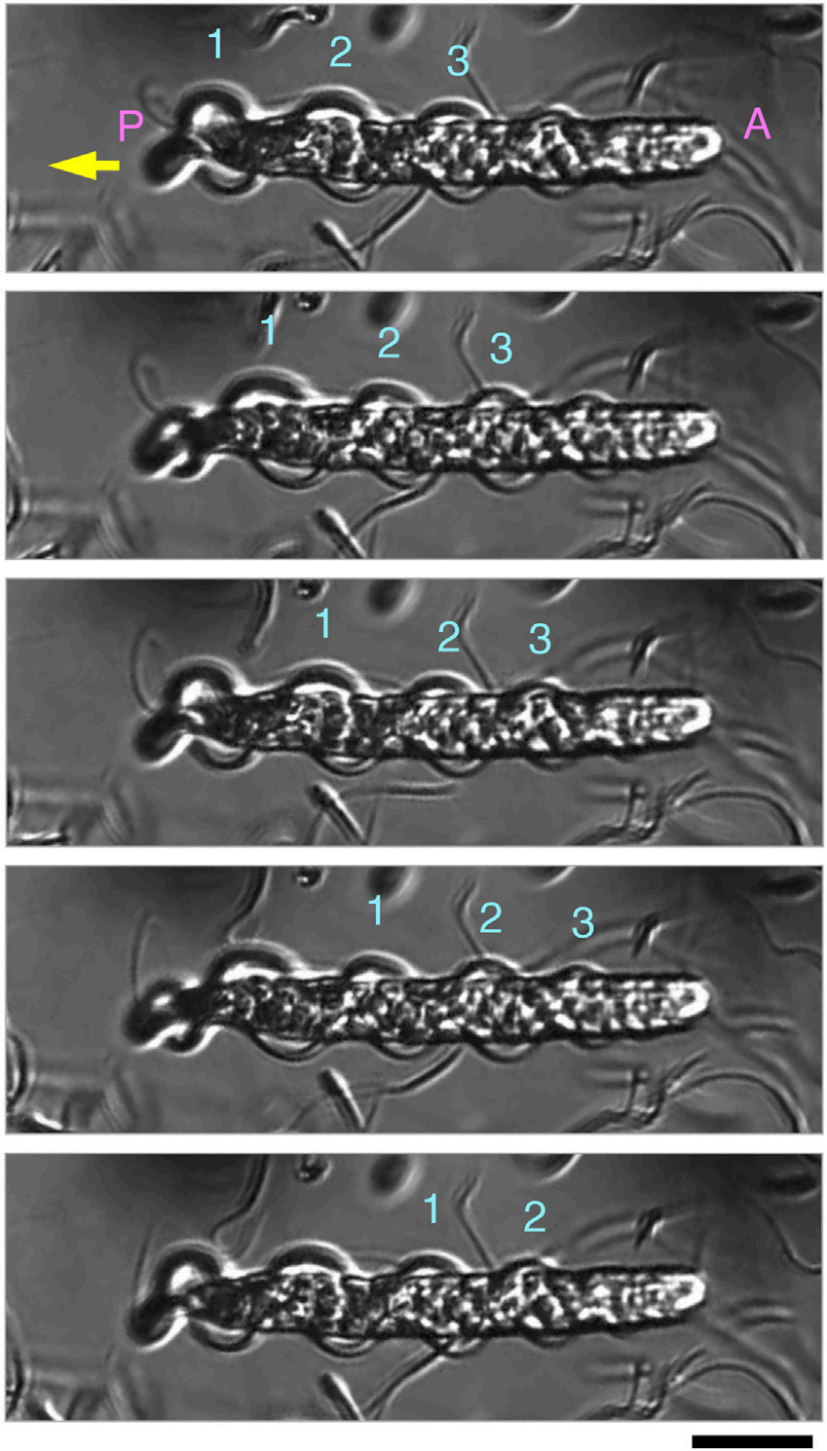
Sequential images of parasperm (top to bottom) recorded at 200 fps in a backward swimming motion (yellow arrow). Images at 15 ms intervals are shown. Three waves (numbers above parasperm) propagated in the undulating membrane from the posterior to anterior end. (A) Anterior end and (P) posterior end. See Supplementary Video S2. Scale bar, 20 μm.

The two undulating membranes propagate flagellar waves for parasperm movement. We evaluated how the direction of movement in the parasperm and wave propagation in the undulating membrane relates to parasperm morphology. Within a few minutes after application onto the glass slide, most mature parasperm were trapped on the surface of the glass slide and transmitted the wave in the forward direction, i.e., from the anterior to the posterior end (Figure 5; Supplementary Videos S1). From observations of parasperm immediately after collection from the sperm duct, some parasperm were capable of rapid backward movement, while others swam relatively slow or were trapped on the surface of the glass slide. Rapid movement was most often observed soon after deposition of the parasperm onto the glass slide and when they swam away from the surface of the glass slide or the surface of the droplet. In these rapidly moving sperm, the wave propagated from the posterior end with a flattened undulating membrane to the anterior end with a round edge (Figure 6; Supplementary Video S2). We analyzed the swimming direction and the propagation of undulating membrane in 46 parasperm from the videos recorded by a high-speed camera. The velocity of forward swimming was 8.0 ± 3.1 μm/s (N = 10), whereas that of backward swimming was 24.5 ± 10.6 μm/s (N = 10). Propagation velocity of anterior-directed undulating membrane was higher than that of posterior-directed (Supplementary Table S1). One parasperm propagated waves both from the anterior and posterior ends (Supplementary Video S3). The propagation velocity of undulating membrane from the posterior end was 34.7 μm/s, whereas that from the anterior end was much higher (408.0 μm/s). This would have resulted in the backward swimming of this parasperm. To examine whether the swimming direction of parasperm is related to the presence of globular bodies, we calculated the percentage of their retainment in each parasperm. Parasperm that retained large or full number of globular bodies appeared to show lower or higher velocity in forward or backward swimming, respectively (Supplementary Table S2), but further analysis will be needed to conclude these correlation.

### Morphological Changes in Paraspermatogenesis

**FIGURE 7:**
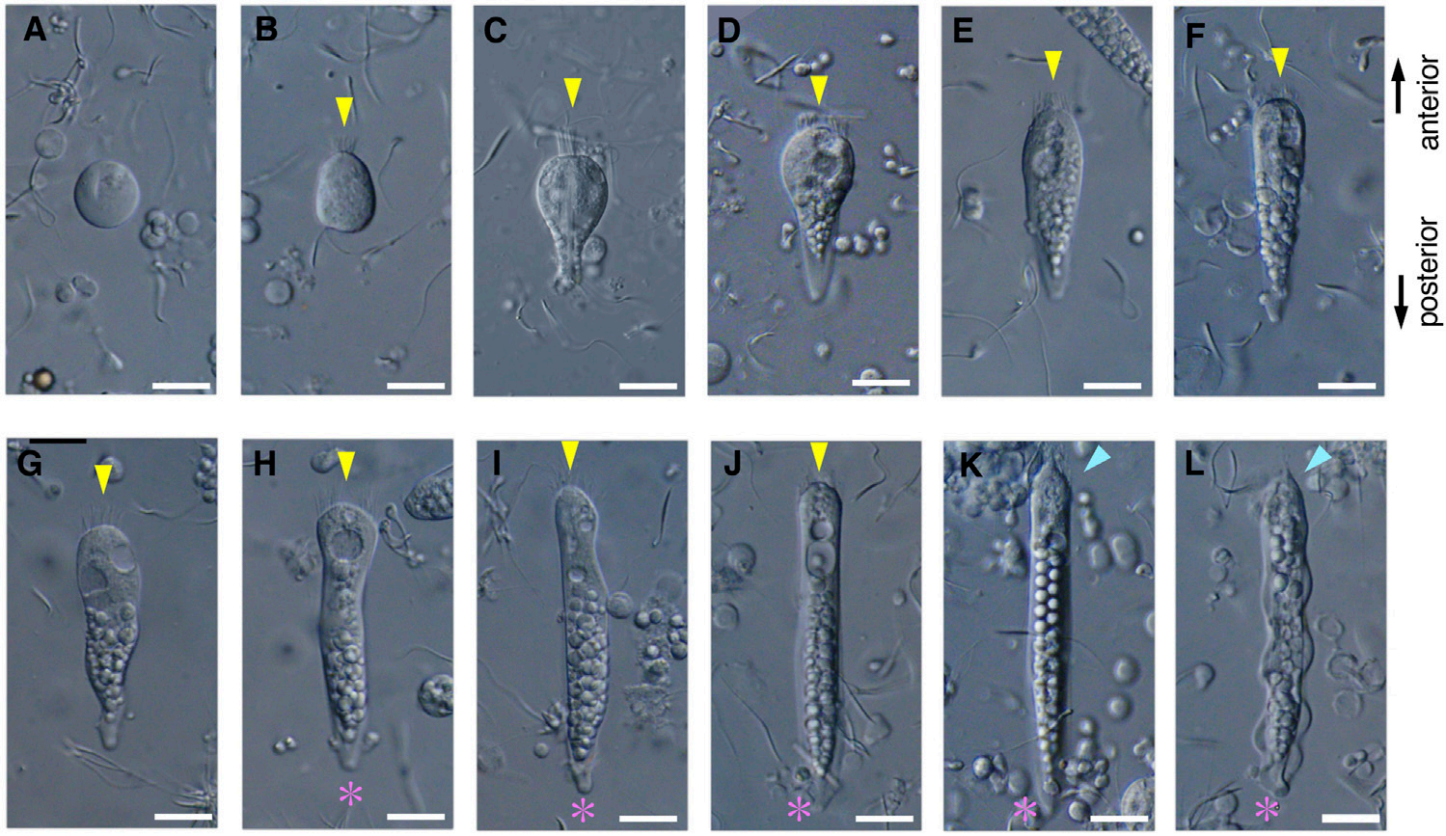
Differential interference contrast (DIC) images of *S. luhuanus* sperm at several stages in spermatogenesis. **(A–C)** Round stage, **(D–G)** cone-shaped stage, and **(H–L)** elongating stage. Yellow arrowheads and magenta asterisks indicate the brush of multiple flagella and the streak of the flattened posterior end, respectively. Blue arrowheads show bundling of the brush in the elongating stage. Scale bar **(A–E)**, 10 μm and **(F–I)**, 5 μm.

**FIGURE 8:**
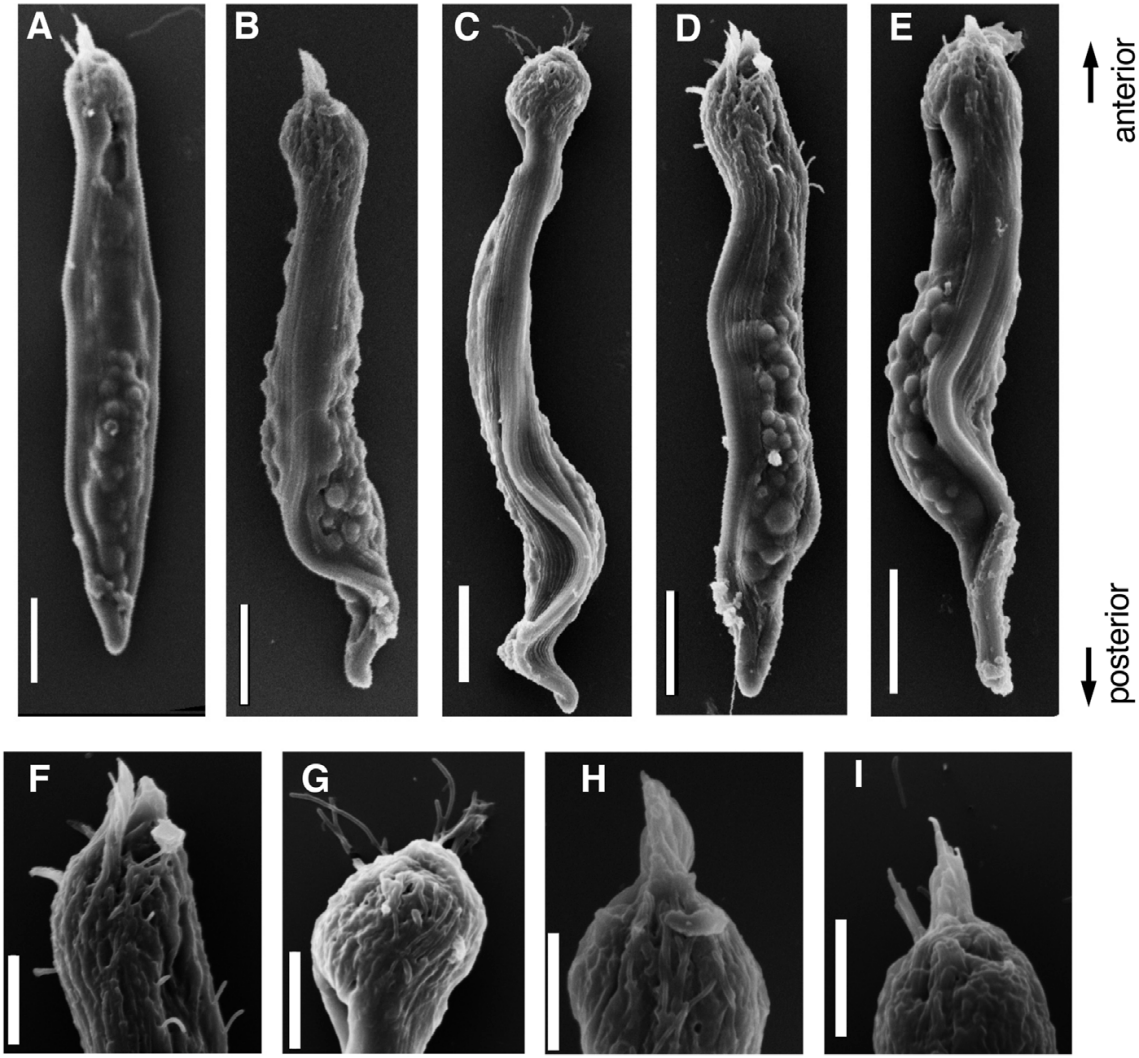
SEM images of **(A–E)** Parasperm at elongating stage. **(F–I)** Magnified images of distinct morphological regions during the elongating stage. The flagellar brushes are bundled at the anterior end. Scale bar **(A–E)**, 10 μm and **(F–I)**, 5 μm.

During the early stages of paraspermatogenesis, the first recognizable cell was spherical with a diameter of approximately 30 μm (Figure 7A). A brush of multiple flagella appeared at the anterior end and later in the cytoplasm (Figures 7B,C). The posterior region extended into a cone shape, and globular bodies started to form in the cytoplasm (Figures 7D–G). Axonemal arrays were observed through the anterior to the posterior end of the cytoplasm. The cell elongated, and the undulating membranes became obvious in the posterior half of the cell when the longer axis reached approximately 100 μm. Finally, the undulating membranes were fully separated from the lateral sides (Figures 7H–L). The flagellar brush at the anterior end could be observed in the late elongating stages (Figure 8); finally, the flagella in this region were bundled into a sharp end (Figures 8F–I). The undulating membrane developed, and the anterior round end of the mature parasperm was filled with globular bodies (Figures 3, 4). Thus, we categorized spermatogenic cells into three stages: round stage (Figures 7A–C), cone-shaped stage (Figures 7D–G), and elongated stage (Figures 7H–L). In this study, we further examined cytoskeletal changes, mainly the behavior of axonemes, in each stage.

### Formation and Dynamics of the Axonemes in Paraspermatogenesis

**FIGURE 9:**
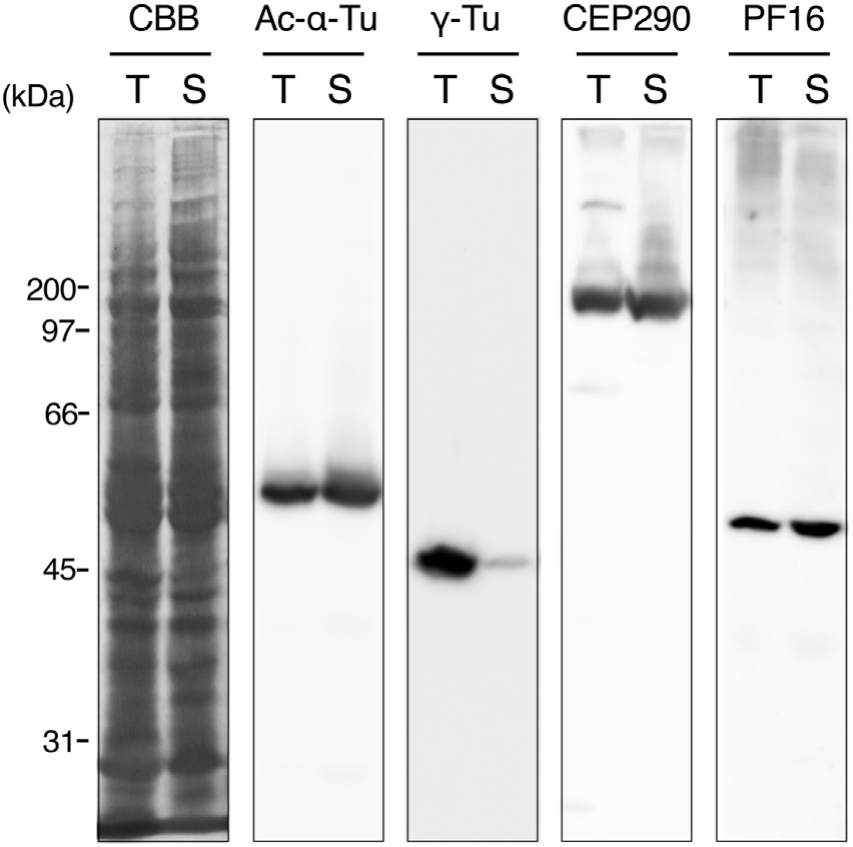
Western blots of whole sperm proteins in testis and sperm duct. The sample from testis or sperm duct contains mixture of immature or mature eusperm and parasperm, respectively. Antibodies for western blotting are indicated at the top. CBB, Coomassie Brilliant Blue R-250 staining. PF16, a component of central pair projection (PF, paralyzed flagella). Note that γ-tubulin is greatly diminished in mature sperm in the sperm duct.

**FIGURE 10:**
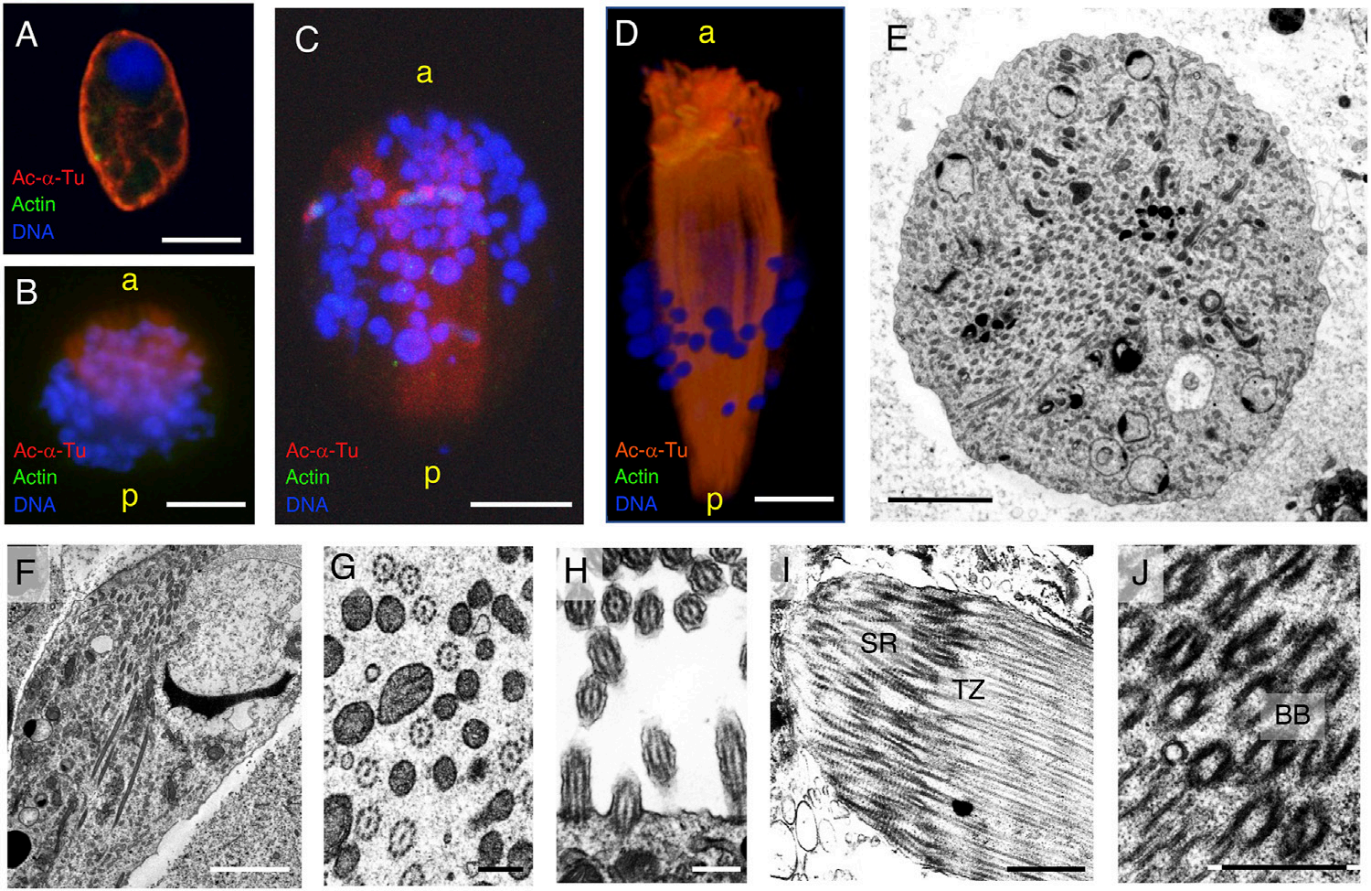
Cytoskeletal structure of the round- and cone-shaped stages of parasperm. Immunofluorescence microscopy was performed for **(A–C)** round stage and **(D)** cone-shape stage parasperm with anti-acetylated α-tubulin (axoneme, red) and anti-actin (cytoplasm, green) antibodies. DNA was stained with DAPI. Bar, 10 μm. **(E–J)** Thin-sectioned TEM image with **(E,F)** a number of axonemes passing through the center of the cytoplasm at the late round stage. **(E)** Cross section, **(F)** semi-longitudinal section. Bar, 5 μm. **(G)** Axoneme structures in the center of the cytoplasm and **(H)** those in the flagellar brushes with single axoneme. Bar, 500 nm. **(I,J)** At anterior region, bundles of the root structure with basal bodies (BB), transition zones (TZ), and striated rootlets (SR) can be observed. Bar, 1 μm.

To describe axonemal behaviors during paraspermatogenesis, we examined the localization of axonemes, centrioles (basal bodies), and transition zones by immunofluorescence using anti-acetylated α-tubulin, anti-γ-tubulin, and anti-CEP290 antibodies, respectively. Western blot analysis showed that γ-tubulin was abundant in immature testicular sperm and scarce in mature sperm (Figure 9), indicating that γ-tubulin, and likely the basal body, was largely lost during spermatogenesis, as is reported for mature sperm in mice (Manandhar et al., 1998) and humans (Fouquet et al., 1998; Fishman et al., 2018). Therefore, the faint band in sperm duct sample appeared due to the residues of basal bodies in mature parasperm (see immunofluorescent analysis below). CEP290, acetylated α-tubulin, and the central apparatus protein PF16 were expressed almost equally in the testis and sperm ducts. In the early round stage, acetylated α-tubulin was first localized in the cytoplasm and the region beneath the plasma membrane (Figure 10A); it then migrated to the proximal half of the cell as well as to the anterior axonemes, at which time the nucleus became fragmented and distributed throughout the cytoplasm, and the brush of the flagella started to extrude (Figure 10B). The axonemes then began to extend toward the inside of the cytoplasm (Figure 10C).

In the cone-shaped stage, the axonemes extended further toward the posterior end and elongated in the brush flagella toward the anterior end (Figure 10D). TEM clearly showed that many 9 + 2 structured axonemes were formed in the cytoplasm without the surrounding plasma membrane (Figures 10E–G), suggesting that they are formed by cytoplasmic ciliogenesis (Avidor-Reiss et al., 2015; Fingerhut and Yamashita, 2020). The axonemes in the anterior brush represent typical cilia structures with 9 + 2 microtubule arrays surrounded by the plasma membrane (Figure 10H). No basal axoneme structures were observed. Later, some brushes showed greater thickness, with multiple cilia extending from the anterior to posterior. Basal bodies and subsequent ciliary rootlets were recognized in the anterior tip of the brush, and transition zones were observed posteriorly from the basal bodies (Figures 10I,J), showing the same direction as the cytoplasmic axonemes.

**FIGURE 11:**
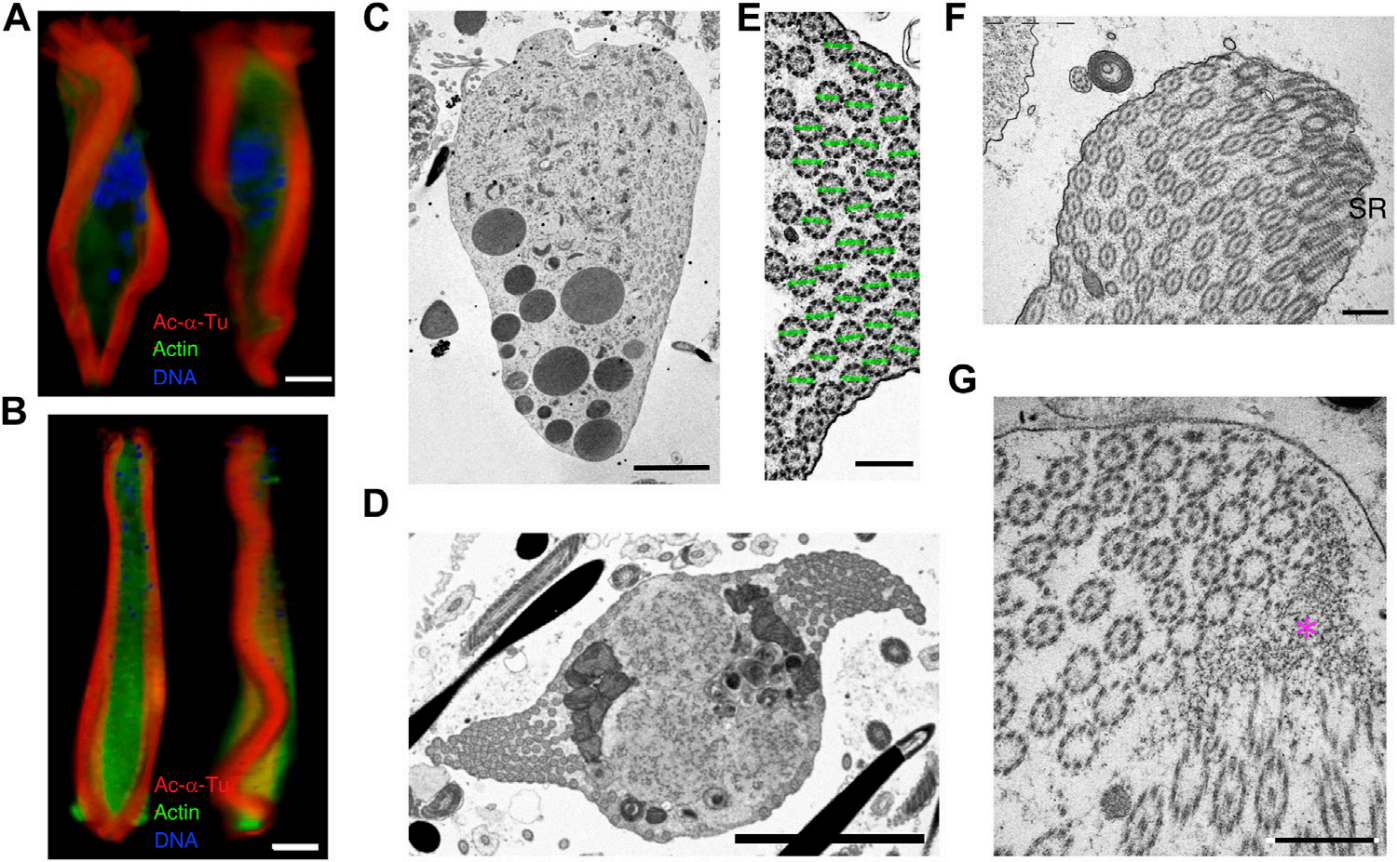
Cytoskeletal structure of the cone-shaped and elongating stage parasperm. Cells were stained with anti-acetylated α-tubulin antibody (axoneme, red), anti-actin antibody (cytoplasm, green), and DAPI. Confocal images from 3D constructed data at different directions for **(A)** early and **(B)** late elongating stage parasperm. Bar, 10 μm. **(C–F)** Thin-sectioned TEM images. **(C)** A longitudinal section, showing the formation of the lateral undulating membrane with the brush at the anterior end. Bar, 5 μm. **(D)** Two undulating membranes are observed on the lateral side of the elongating parasperm. Bar, 5 μm. **(E)** Magnified image of the axonemes in the undulating membrane, showing alignment of axonemal direction. Green lines represent the plane of two singlet microtubules. Bar, 500 nm. **(F–G)** Structures at the **(F)** anterior and **(G)** posterior ends of an elongating parasperm. ST, striated rootlet. Magenta asterisk, electron dense materials. Scale bar, 500 nm.

In the elongated state, the fragmentation of the nucleus progressed (Figures 11A,B), and the mass of axonemes in the center of the cytoplasm was separated into two lateral sides, forming undulating membranes. At the posterior end, the undulating membranes were joined, though they were still separated by brush structures at the anterior end (Figures 11A–D). The axonemes in the undulating membrane were aligned with the planes of the central pair of microtubules, almost parallel to the lateral direction (Figure 11E). Basal structures of the axonemes were observed at the anterior end (Figure 11F). At the posterior end, axonemes were anchored to electron-dense materials near the plasma membrane, where the axonemes showed 9 + 0 structures (Figure 11G).

**FIGURE 12:**
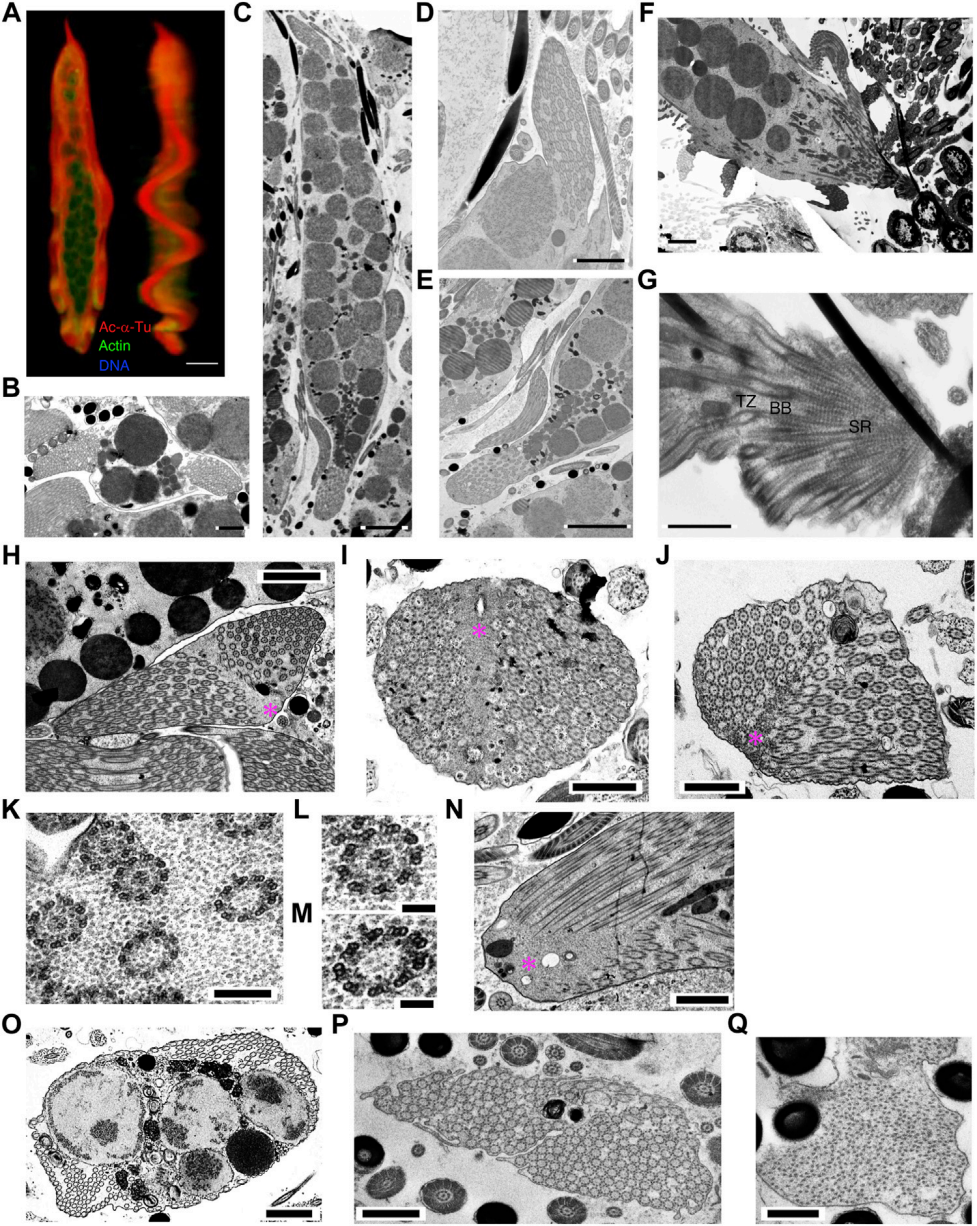
Cytoskeletal structure of mature parasperm. Cells were stained with anti-acetylated α-tubulin antibody (axoneme, red), anti-actin antibody (cytoplasm, green), and DAPI. **(A)** A front and lateral confocal image from 3D constructed data. Bar, 10 μm. **(B–O)** Thin-sectioned TEM images. **(B)** A cross and **(C)** a longitudinal section of a mature parasperm. **(B)** Two lateral undulating membranes and **(C)** both anterior and posterior end structures can be observed. Bar, 2 μm **(B)**; 5 μm **(C)**. Magnified images of **(D)** anterior and **(E)** posterior ends. Bar, 2 μm **(D)**; 5 μm **(E)**. **(F)** A longitudinal section of a parasperm showing a prominent edge at the anterior end. **(G)** Magnification of **(F)** showing that the projection at the anterior end is composed of basal bodies and striated rootlets. Bar, 2 μm **(F)**; 1 μm **(G)**. **(H–J)** Axonemal arrangements at the posterior end. Electron dense materials are seen as a streak in the center of the posterior end, where two arrays of axonemes are joined, with 9 + 0 structured axonemes frequently observed near the materials. Bar, 2 μm **(H)**; 1 μm **(I,J)**. **(K–M)** Magnification of the axonemes shows 9 + 2 structurers in the lateral region and 9 + 0 structures with both outer and inner arms located centrally near the dense materials. Bar, 200 nm **(K)**; 100 nm **(L,M)**. **(N)** A longitudinal section of the posterior end. Bar, 1 μm. **(O–Q)** A number of arrays of the axonemes with 9 + 0 structure are occasionally observed in the **(O)** lateral undulating membranes and **(P)** posterior end. Bar, 1 μm. **(Q)** The tip of the axonemes shows a collapsed structure. Bar, 500 nm.

In mature parasperm, the brush structures finally joined at the anterior end of the two lateral undulating membranes (Figures 12A–E). All axonemes were separated into undulating membranes, except those lined through the surface of globular bodies (Figures 4I–K). The number of axonemes in the undulating membrane of mature parasperm were 142.5 ± 26.5 (N = 12). Considering that mature parasperm are generated as syncytia without cytokinesis, this number of the axonemes would be equivalent to that predicted from seven or eight divisions of paraspermatogonia.

A 9 + 0 structured region was observed in the transition zones at the anterior end (Figures 12F,G). These structures were not only observed in mature parasperm, but also near the basal body/striated rootlet region in the cone-shaped stage (Figure 11F). In the posterior region, the distal ends of the axonemes were anchored to electron-dense materials (Figures 12H–J), as observed in the cone-shaped stage (Figure 11G). Electron-dense materials were extended through this region, observed as a central streak, where arrays of axonemes from both lateral sides are joined (Figures 12H–J). Axonemes with 9 + 0 structure were often observed near the joint of two lateral axonemal arrays. These parts of the axonemes possessed both outer and inner arms and were therefore not considered part of the axonemal transition zones at their bases (Figures 12K–M). Several studies have reported a 9 + 0 structure without dynein arms at the transition zone (Fisch and Dupuis-Williams, 2011; Czarnecki and Shah, 2012). Intriguingly, in mature parasperm, arrays of 9 + 0 axonemes with dynein arms were frequently observed in the lateral undulating membranes at the posterior end and near a mass of globular bodies (Figures 12O–P). The 9 + 0 axonemal structures showed partially degenerated at the posterior end (Figure 12Q). These axonemes appeared to have dynein arms, suggesting that the structure observed at the posterior end would be the distal tip of the axoneme.

### Changes in the Distribution of γ-Tubulin and CEP290

**FIGURE 13:**
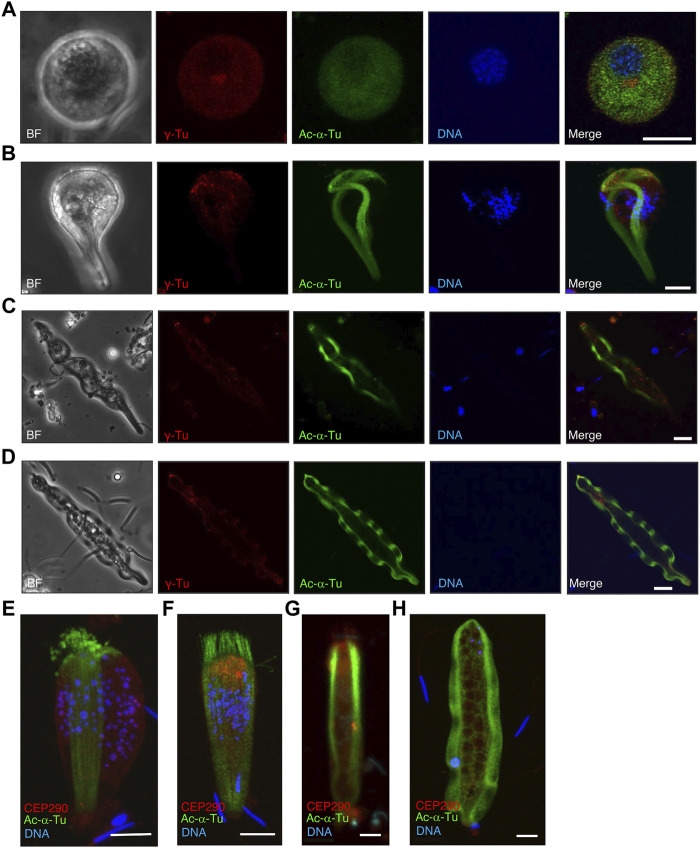
Localization of γ-tubulin and CEP290 in several stages of paraspermatogenesis. Several stages of parasperm were stained with anti-acetylated α-tubulin antibody (green), anti-γ-tubulin antibody (**A–D**, red), anti-CEP290 antibody (**E–H**, red), and DAPI. **(A)** γ-Tubulin first accumulates in the anterior region at the late round stage and then **(B–D)** decreases and becomes localized at the tip of anterior end. BF, bright field image. Bar, 10 μm. **(E,F)** CEP290 accumulates in the anterior region in the cone-shaped stage, **(G)** but its localization becomes obscure, and **(H)** is no longer apparent in mature parasperm. Bar, 10 μm.

Western blotting showed that γ-tubulin was largely lost during spermatogenesis in *S. luhuanus*. To determine the changes in the basal structures, we assessed the distribution of γ-tubulin and CEP290, which are known to be located in the basal body (centrioles) and transition zone, respectively. In the early round stage of the parasperm lacking the flagellar brush in the posterior region, centrioles were observed near the nucleus (Figure 13A). Later, when the brush was formed and the nucleus started to fragment, γ-tubulin multiplied, migrated, and accumulated in the anterior region at the late round stage (Figure 13B); it then decreased and became localized at the tip of the anterior end (Figures 13C,D). In contrast, CEP290 was almost evenly distributed in the cytoplasm (Figure 13E), but its mass emerged in the anterior region during the extension of the flagellar brushes and cytoplasmic axonemes (Figure 13F). In the elongating state, CEP290 was distributed throughout the parasperm and was slightly more concentrated in the anterior region, including in the flagellar brush (Figure 13G). In mature parasperm, CEP290 was sparsely distributed in the cytoplasm (Figure 13H).

## Discussion

*Strombus* snails have been reported to produce unique parasperm phenotypes. However, their cellular structures and motile characteristics are largely unknown. Here, we describe the motility of the parasperm in relationship to its cytoskeletal structure. Reports on the orientation of *Strombus* parasperm have been controversial. Most studies have recognized the round end as the anterior end (Nishiwaki, 1964; Koike, 1985; Koike and Shinohara, 1987; Buckland-Nicks, 1998); however, the brush structure at the anterior end of elongating parasperm was observed at the posterior end by Buckland-Nicks (1998). In *Fusitriton* parasperm, the anterior end is anchored by the rootlet from the flagellar basal body; therefore, the axonemal tip is positioned at the posterior region (Buckland-Nicks et al., 1982). Similarly, in *Strombus* we observed a group of axonemes with 9 + 0 structures located around the streak area in the posterior region. The characteristics of the regions with a 9 + 0 structure and their specific configuration at the posterior end need further clarification.

The 9 + 0 structure that we observed at the posterior end was previously recognized as the basal body at the anterior end (Koike and Nishiwaki, 1980). However, we confirmed that the basal bodies were localized in the flagellar brush at the anterior end, not at the posterior end. This is supported by the fact that the transition zone protein, CEP290, was localized at the anterior end or in the flagellar brush. The 9 + 0 structure at the streaked end (at the posterior end) could not be the basal body given the presence of doublet microtubules with both outer and inner arms, and the tips of the axonemes having been disorganized. We observed some 9 + 0 axonemes with dynein arms in the middle of the streaked end, where axonemes from the two undulating membranes would join and fold together. The axonemes in this section are thought to be highly flexible and deformed. However, it remains unclear how the two lateral undulating membranes are attached or fused at the posterior end and how the 9 + 0 structure is linked to wave propagation.

We previously reported that eusperm change motility in seawater autonomously, including backward asymmetric movement for detachment from the sperm bundle, circular and straight forward movement, and spontaneous backward swimming with symmetric flagellar waveforms (Shiba et al., 2014). As Nishiwaki (1964) briefly mentioned, the *Strombus* parasperm also exhibited backward swimming. In the present study, we found that parasperm often showed backward swimming near the surface of seawater or during free swimming and reversed the swimming direction when they were attached or near the glass surface. Swimming velocity was much higher in backward swimming than in forward swimming. These changes in direction and velocity are related to the physiological function of the parasperm, from ejaculation to migration through the female reproductive tract to the sperm receptacle. Although the physiological functions of parasperm have been experimentally described in a few cases (Buckland-Nicks, 1998; Hayakawa, 2007), the intrinsic characteristics of parasperm motility also reflect their *in vivo* behavior and function in internal fertilization and reproductive strategies. We previously reported that eusperm use backward movement when they are detached from the wall of sperm receptacle (Shiba et al., 2014). One possible idea for the roles of *Strombus* parasperm is that they are involved in sperm competition. Their backward movement might block and interfere with the movement of eusperm from other males. Furthermore, we observed that the globular bodies of some parasperm are lost, suggesting that these bodies might be released during fertilization process and play some roles such as provision of nutrient for self-derived eusperm or agglutination of eusperm from other males. Future research should consider how pertinent environmental factors affect parasperm motility. For example, it would be intriguing to assess whether changes in extracellular Ca^2+^ would affect the direction of swimming in parasperm, as observed in eusperm (Shiba et al., 2014).

The formation of eukaryotic cilia and flagella depends on the membrane-mediated transport of their components, i.e., intraflagellar transport (Rosenbaum and Witman, 2002; Scholey, 2008). A large number of axonemes form and extend in the cytoplasm during paraspermatogenesis in several snail species, including *Strombus* (Buckland-Nicks et al., 1982; Buckland-Nicks, 1998; Gimenez, 2011). Given that the dependency on intraflagellar transport differs between sperm flagella and somatic cilia (Han et al., 2003; Sarpal et al., 2003), another process for axonemal formation, i.e., cytoplasmic or cytosolic ciliogenesis, emerged in Drosophila (Briggs et al., 2004; Avidor-Reiss and Leroux, 2015; Fingerhut and Yamashita, 2020) and Plasmodium sperm (Sinden et al., 2010). Although this process was described more than 40 years ago, its mechanism remains unclear (Fawcett et al., 1970; Tates, 1971; Tokuyasu, 1975; Sinden et al., 1976).

Cytoplasmic formation of multiple axonemes was reported in prosobranch parasperm more than a century ago (Reinke, 1912). Multiple axonemes are formed by the multiplication of centrioles followed by their attachment to the plasma membrane (Gall, 1961; Tochimoto, 1967; Nishiwaki and Tochimoto, 1969; Healy and Jamieson, 1981; Buckland-Nicks, 1998). Numerous basal bodies, i.e., centrioles at the base of cilia and flagella, were observed during the early stages of paraspermatogenesis. The direction of axonemal extension was completely reversed in paraspermatogenesis compared to intraflagellar transport-dependent ciliogenesis, as observed in Chlamydomonas flagella and epithelial cilia. The striated rootlets were connected to the basal bodies and directed to the plasma membrane (Figures 11, 12; Buckland-Nicks et al., 1982). Axonemal extension occurred toward the center of the cytoplasm from the 9 + 0 structured transition zone, which was formed from the basal body. We did not observe any basal bodies at the brushed anterior end of the parasperm, according to western blotting with anti-γ-tubulin antibody.

The transition zone functions as a barrier or gate for the transport of ciliary components (Reiter et al., 2012) and contributes to axonemal formation in Drosophila spermatogenesis (Vieillard et al., 2016). CEP290, a major component of the transition zone, mediates binding between doublet microtubules and the plasma membrane (Craige et al., 2010). In *Strombus* paraspermatogenesis, transition zones were observed in the brushed areas as well as in the main cytoplasm adjacent to the globular bodies near the anterior end. Therefore, the transition zone is considered not only to function in the transport of axonemal components, as previously reported, but would play another role in further axonemal elongation in the cytoplasm.

The arrangement and the configuration of undulating membranes would be sophisticated enough to bring out the production of planar waves for parasperm swimming. In the comb plates of ctenophores, doublet microtubules are physically linked at positions 3 and 8 between adjacent cilia through a structure called compartmenting lamella (Jokura et al., 2019). The structure ensures concerted orientation among tens of thousands of cilia for the coordinated planar movement. However, no apparent structure that links multiple axonemes is observed in the undulation membrane of parasperm. In multiciliated cells, the direction of beating is regulated by both the orientation of basal bodies and the mechanical feedback of hydrodynamic forces (Mitchell et al., 2007). Since the axonemes are elongated over in the undulating membrane with more than ∼100 μm in length, it is likely that the hydrodynamic forces generated by coordinated movement of multiple axonemes would much contribute to the planar wave propagation.

It is not clear how the waves could be initiated from both anterior and posterior ends, or how 9 + 0 structured part of the axonemes contributes to the wave propagation. In conclusion, we characterized the motility of parasperm in the marine snail *S. luhuanus* according to the properties of its axonemal arrays. We propose that parasperm motility, particularly the reversion of swimming direction, is connected to cell behavior in the female reproductive tract. The present study sheds light not only on the mechanism of paraspermatogenesis but also on the cytoplasmic formation of axonemes and the loss of centrioles.

## Data Availability

The original contributions presented in the study are included in the article/Supplementary Material, further inquiries can be directed to the corresponding author.

## Supplementary Material

Supplementary Video S1 Swimming of mature parasperm by wave propagation in the undulating membrane (forward movement). The movie was recorded at 200 fps and analyzed at real-time (0–5.28 s) and 0.15 ×) and –10.00 s) speed respectively. Scale bar, 20 μm.

Supplementary Video S2 Swimming of mature parasperm by wave propagation in the undulating membrane (backward movement). The movie was recorded at 200 fps and analyzed at real-time (0–2.06 s) and 0.15 × (2.07–10.00 s) speed, respectively. Scale bar, 20 μm.

Supplementary Video S3 The movement of parasperm showing wave propagation by the undulating membranes in both directions. The movie was recorded at 200 fps and analyzed at 0.15 × speed. The waves from the anterior and posterior ends propagate toward the posterior and anterior sides, respectively (indicated by yellow arrows). The anteriorward waves are more rapidly propagated than the posteriorward waves. Scale bar, 20 μm.

## Abbreviations

CEP290, centrosomal protein 290; DIC, differential interference contrast; TEM, transmission electron microscopy; SEM, scanning electron microscopy.
